# Inter-method and anatomical correlates of episodic memory tests in the Alzheimer’s Disease spectrum

**DOI:** 10.1371/journal.pone.0223731

**Published:** 2019-10-10

**Authors:** Felipe Kenji Sudo, Andrea Silveira de Souza, Claudia Drummond, Naima Assuncao, Alina Teldeschi, Natalia Oliveira, Fernanda Rodrigues, Gustavo Santiago-Bravo, Victor Calil, Gabriel Lima, Pilar Erthal, Gabriel Bernardes, Marina Monteiro, Fernanda Tovar-Moll, Paulo Mattos

**Affiliations:** 1 D’Or Institute for Research and Education, Rio de Janeiro, RJ, Brazil; 2 Department of Speech and Hearing Pathology, Federal University of Rio de Janeiro, Rio de Janeiro, RJ, Brazil; 3 Morphological Sciences Program, Institute of Biomedical Sciences, Federal University of Rio de Janeiro, Rio de Janeiro, RJ, Brazil; 4 Post-Graduation Program in Clinical Medicine, Faculty of Medicine, Federal University of Rio de Janeiro, Rio de Janeiro, RJ, Brazil; 5 Institute of Psychiatry, Federal University of Rio de Janeiro, Rio de Janeiro, RJ, Brazil; Nathan S Kline Institute, UNITED STATES

## Abstract

**Background:**

Episodic memory impairments have been described as initial clinical findings in the Alzheimer’s Disease (AD) spectrum, which could be associated with the presence of early hippocampal dysfunction. However, correlates between performances in neuropsychological tests and hippocampal volumes in AD were inconclusive in the literature. Divergent methods to assess episodic memory have been depicted as a major source of heterogeneity across studies.

**Methods:**

We examined correlates among performances in three different delayed-recall tasks (Rey-Auditory Verbal-Learning Test–RAVLT, Logical Memory and Visual Reproduction subtests from the Wechsler Memory Scale) and fully-automated volumetric measurements of the hippocampus (estimated using Neuroquant^®^) of 83 older subjects (47 controls, 27 Mild Cognitive Impairment individuals and 9 participants with Dementia due to AD).

**Results:**

Inter-method correlations of episodic memory performances were at most moderate. Scores in the RAVLT predicted up to 48% of variance in HOC (Hippocampal Occupancy Score) among subjects in the AD spectrum.

**Discussion:**

Tests using different stimuli (verbal or visual) and presenting distinct designs (word list, story or figure learning) may assess divergent aspects in episodic memory, with heterogeneous anatomical correlates.

**Conclusions:**

Different episodic memory tests might not assess the same construct and should not be used interchangeably. Scores in RAVLT may correlate with the presence of neurodegeneration in AD.

## Introduction

Episodic memory refers to human’s ability to consciously recollect situations and events through the effective acquisition, retention and recall of verbal and visuospatial data[1]. From a neural perspective, a rich body of evidence concerning the pivotal role of the hippocampus for the space-time organization and storage of information has been provided by studies with geriatric population, especially those focused on the Alzheimer’s Disease (AD) spectrum[2]. In those cases, it has become accepted that hippocampal dysfunction occurs during the course of the disorder, following many years of asymptomatic parenchymal accumulation of β-amyloid peptides and tau protein[3]. With the escalation of this neuropathological process, the clinical stage of the disorder initiates, typically manifesting as early episodic memory impairments[3,4].

Regarding the current knowledge about the pathophysiology of AD, episodic memory testing and structural neuroimaging remain relevant for the assessment of older subjects with suspected cognitive impairment[5]. However, findings on the correlates between cognitive and AD-related anatomical features are largely discordant, which might be attributed to the complex nature of episodic memory itself, as well as to inconsistencies across methods for neuropsychological assessment[4] and brain volume estimation[6,7]. For instance, episodic memory tasks may apply stimuli of different materials (verbal or visuospatial, for example) or they may assess distinct components within this cognitive ability (acquisition, retention and delayed-recall)[4]. Moreover, measurement models range from evaluating memory capacity for semantically uncorrelated items (verbal item-memory tasks, such as word lists[8]) to investigating recollection of sequences of logically-linked ideas (for example, story learning)[9]. Thus, discrepant episodic memory performances across studies could be interpreted as effects of samples with different levels of cognitive impairment or as influence of disproportional task-specific demands[10,11].

Likewise, an array of strategies has been described in the literature for the evaluation of medial temporal cortex atrophy in AD, such as visual rating scales and computer-based methods, namely manual, semi-automated and automated volumetric measurements[12]. Translating brain volumetrics into clinical practice has been hampered by many factors, such as the high cost of the instruments, the time-consuming processing operations, the lack of harmonized approaches across laboratories and the paucity of normative data for grey and white matter volumes among older population [11,13,14]. As an effort to overcome those limitations, NeuroQuant^®^, an FDA approved software for automatic labeling, visualization and volumetric quantification of brain structures, was commercially released by CorTechs Laboratories in 2007. This method has been cross-validated with manual segmentation[15] and with other well-known brain morphometry procedures in AD samples (FreeSurfer, for example)[16]. In addition, neuroimaging parameters in NeuroQuant^®^ for each subject have been compared to an extensive and continuously growing cloud-based normative database[17].

Analyzing correlations across different episodic memory tasks would allow inferring about whether those instruments could be employed interchangeably. Specifically, it would clarify about the construct validity of those neuropsychological tests. In addition, according to studies, hippocampal volume appears to be the strongest individual predictor of short-term cognitive decline in older population, compared to other AD biomarkers[18]. Estimating associations between scores in memory tests and brain volumes could indicate which cognitive index best reflects neurodegeneration in subjects within the AD spectrum. Therefore, the present study aimed at investigating: (1) the convergent validity of different measures of episodic memory; and (2) the clinical-anatomical correlation between memory performances and volumetric indices in normal older controls and individuals in the AD continuum.

## Methods

### Participants

The present study is a branch of a larger longitudinal study on cognitive impairment in the Brazilian population conducted in the D'Or Institute of Research and Education and the Federal University of Rio de Janeiro since 2011. Eligible participants were non-institutionalized older adults (>55 years old), with 7 or more years of schooling, native Brazilian Portuguese speakers, presenting memory complaints. Exclusion criteria were as follows: current major depressive disorder (according to the 5th edition of the Diagnostic and Statistical Manual of Mental Disorders—DSM-5)[5]; current delirium[5]; history of severe psychiatric disorders (e.g., schizophrenia, intellectual disability, bipolar disorder)[5]; history of substance-related disorders[5]; severe visual or auditory impairments that precluded neuropsychological testing; refusal to complete the neuropsychological and neuroimaging protocol; and contraindications to brain MRI (e.g., ferromagnetic intracranial aneurysm clips or cardiac pacemaker).

### Procedures

Initially, participants were submitted to a clinical interview by a physician, followed by physical examination, to verify the eligibility for the study. Subsequently, a neuropsychologist administered the Brazilian versions of the following instruments: the Mini-Mental State Examination[19], the Rey-Auditory Verbal-Learning test (RAVLT)[20], the Logical Memory and the Visual Reproduction subtests of the Wechsler Memory Scale[21]. Verbal item-memory evaluation encompassed scores in RAVLT A5, which correspond to the number of acquired information in the last trial of the learning phase of the test, and in RAVLT A7, which refer to the 30-minute delayed recall trial[20]. Moreover, raw values of recollected items in Logical Memory and Visual Reproduction tests were used in this analysis.

Behavioral and functional assessment questionnaires comprised: the Neuropsychiatric Inventory[22], the Geriatric Depression Scale[23] and Lawton-Brody Instrumental Activities of Daily Living Scale[24].

Participants underwent an image acquisition protocol in a 3T magnetic resonance scanner (Achieva, Philips Medical Systems) including an isotropic high-resolution 3D T1-weighted sequence (TR/TE 13/ 1.4 ms; matrix 256 x 256 mm; FOV 240 mm; slice thickness 1 mm; 140 slices). Trained radiologist and medical physicists blinded to all evaluations analyzed the images for potential exclusion criteria. Moreover, visual assessment of images for potential hippocampal atrophy was conducted[13].

Raw T1-weighted DICOM sequences were processed by the Neuroquant^®^ software–version 2 package. The algorithm used in this procedure has been previously detailed[15] and it encompasses the following stages: (i) quality assessment of structural MRI data for artifacts; (ii) gradient and B1 field corrections; and (iii) automated segmentation of brain regions-of-interest (ROIs), based on the neuroanatomical label attributed to each voxel within the targeted structure by a probabilistic atlas. Output of this method includes volumetric data (in cubic centimeters and percentage of intracranial volume—ICV) and images with each segmental structure marked in a specific color. ICV-corrected volumes of the total cortical grey matter (CGM), hippocampi and inferior lateral ventricles (ILV) from each hemisphere were extracted. In addition, the Hippocampal Occupancy Score (HOC) was automatically calculated, using the following equation: (left hippocampal volume / left hippocampal volume + left inferior lateral ventricle volume) + (right hippocampal volume / right hippocampal volume + right inferior lateral ventricle volume)[25].

### Diagnoses

Participants were classified as normal controls (NC), Mild Cognitive Impairment due to AD (MCI) and dementia due to AD (DAD) using the 2011 National Institute on Aging-Alzheimer’s Association criteria[26,27]. For this purpose, analyses of clinical and neuropsychological data, as well as a visual inspection of MRI, were conducted and integrated by the whole multidisciplinary team. Evidence of AD pathology was determined by the presence of hippocampal atrophy using MTA visual assessment[13].

### Ethical standards

All the participants provided a written informed consent prior to the inclusion in the study. Capacity to provide consent to participation in the research was determined during the initial interview and the following principles were adopted: (i) persons with cognitive impairment or dementia were presumed to have the capacity to consent unless established otherwise; (ii) regardless of their cognitive status, the participants’ preferences regarding their inclusion on the research were guaranteed; and (iii) since no structured instrument for consent assessment is available for older Brazilian population, a qualitative assessment was conducted and decisions to include or not the volunteer were based on the clinician’s impression about the subjects’ understanding and reasoning capacities[28,29].

The project was approved by the Research Ethics Committee of the D’Or Institute under the protocol no. 226/11. The principles of the Resolution n. 510/2016 of the Brazil’s National Health Council, which regulates research involving human beings in the country were followed. In addition, the authors assert that all procedures contributing to this work comply with the ethical standards of the Helsinki Declaration of 1975, as revised in 2008.

### Statistical analysis

Data was checked for parametric assumptions based on visual inspection of histograms and values of skewness and kurtosis <1.96[30]. Mean differences in continuous data were compared across diagnostic groups using one-way analysis of variance (ANOVA), for normally distributed variable, or Kruskal-Wallis test, for the other cases. Welch’s test was applied for normally distributed data that violated the assumption of homogeneity of variance. Dunnett’s T3 post-hoc test was used to allow adequate pairwise comparisons, considering that groups showed unequal and small sample sizes [31,32]. Alternatively, when significant group differences were detected in Kruskal-Wallis test, serial Mann-Whitney tests were conducted to detect pairwise distinct medians. Distribution of sex among groups were analyzed using Pearson’s Chi Square. To assess convergent validity within episodic memory domain, partial correlations among the three memory tests were tested, adjusting for age and schooling. In addition, correlations between memory tasks and brain volumes were investigated. Analyses were conducted for the whole sample and for subjects with MCI and DAD. Value of α was adjusted for multiple comparisons and was set at p< 0.005 for all correlations. Due to unequal sample sizes across groups, correlation analyses were conducted using the whole sample and a combination of MCI+DAD participants. In addition, minimum correlation coefficient was corrected for the sample sizes, for a power of 80%, as follows: for the whole sample, lowest sizeable correlation was established as r = 0.4 to 0.5; whereas for analysis restricted to the MCI+DAD, moderate correlations corresponded to 0.5<r<0.7[28]. Rule-of-thumb for interpreting size of correlation coefficients were adopted, as depicted in previous studies[33]. Correlations between RAVLT and HOC were plotted. For all calculations, the IBM Statistical Package for the Social Sciences v. 25 (IBM Corp. IBM SPSS Statistics for Windows, Version 25.0. Armonk, NY: IBM Corp.) was used.

## Results

### Descriptive analyses

A total of 83 subjects were included in the study and were classified into three groups: NC (n = 47), MCI (n = 27) and DAD (n = 9). Participants in the NC group were significantly younger and presented more years of schooling than MCI subjects (p<0.001 and p = 0.001, respectively). No significant differences regarding sex distribution across diagnostic categories was found (p = 0.33). Performances in the MMSE and in all memory tasks distinguished the three diagnostic groups (p<0.001 for all comparisons). Adjusting for intracranial volumes, bilateral hippocampal and ILV volumes, as well as HOC and right CGM, differentiated the three groups (p<0.001 for all analyses), whereas left CGM volumes were only significant when comparing controls with MCI and DAD (p<0.001). Table 1 depicts those results.

**Table 1:** Sociodemographic characteristics, mean scores in cognitive assessment and brain volumes, corrected for intracranial volumes of the sample.

Variables	All	NC	MCI	DAD	p-value	Contrasts
**n**	83	47	27	9	-	-
**Age** (years), mean (SD)	70.07 (7.08)	67.36 (6.36)	72.85 (4.94)	75.88 (8.62)	< .001	NC≠MCI
**Schooling** (years), mean (SD)	14.57 (2.59)	15.61 (1.54)	12.85 (2.82)	14.33 (3.60)	.001*	NC≠MCI
**Sex** (% female)	66.3%	72.3%	55.6%	66.7%	.33	-
**MMSE**, mean (SD)	26.71 (2.60)	27.97 (1.51)	26.07 (1.87)	22.00 (3.04)	< .001**	NC≠MCI; NC≠DAD; MCI≠DAD
**RAVLT** A5, mean (SD)	10.54 (2.86)	12.19 (1.72)	9.11 (2.53)	6.22 (1.85)	< .001	NC≠MCI; NC≠DAD; MCI≠DAD
**RAVLT** A7, mean (SD)	7.68 (3.99)	10.02 (2.42)	5.46 (3.57)	2.11 (1.96)	< .001	NC≠MCI; NC≠DAD; MCI≠DAD
**Logical Memory**, mean (SD)	17.82 (9.63)	22.86 (6.88)	13.92 (8.79)	3.33 (2.29)	< .001*	NC≠MCI; NC≠DAD; MCI≠DAD
**Visual reproduction**, mean (SD)	35.22 (25.67)	47.58 (22.64)	24.57 (20.09)	2.77 (4.17)	< .001*	NC≠MCI; NC≠DAD; MCI≠DAD
**Left CGM** (%ICV), mean (SD)	13.57 (1.04)	13.92 (1.08)	13.30 (0.67)	12.54 (0.84)	< .001	NC≠MCI; NC≠DAD
**Right CGM** (%ICV), mean (SD)	13.66 (1.08)	14.08 (1.04)	13.39 (0.66)	12.31 (1.00)	< .001	NC≠MCI; NC≠DAD; MCI≠DAD
**Left Hippocampus** (%ICV), mean (SD)	0.21 (0.03)	0.46 (0.06)	0.41 (0.05)	0.33 (0.06)	< .001	NC≠MCI; NC≠DAD; MCI≠DAD
**Right Hippocampus** (%ICV), mean (SD)	0.22 (0.04)	0.23 (0.03)	0.21 (0.02)	0.15 (0.02)	< .001	NC≠MCI; NC≠DAD; MCI≠DAD
**Left ILV** (%ICV), mean (SD)	0.08 (0.04)	0.06 (0.02)	0.08 (0.02)	0.16 (0.09)	< .001*	NC≠MCI; NC≠DAD; MCI≠DAD
**Right ILV** (%ICV), mean (SD)	0.08 (0.04)	0.06 (0.02)	0.08 (0.03)	0.16 (0.02)	< .001*	NC≠MCI; NC≠DAD; MCI≠DAD
**HOC**, mean (SD)	0.72 (0.12)	0.78 (0.08)	0.69 (0.09)	0.51 (0.09)	< .001	NC≠MCI; NC≠DAD; MCI≠DAD

*Welch’s ANOVA

**Kruskal-Wallis test; n = sample size; SD = Standard Deviation; MMSE = Mini-Mental State Examination; NC = Normal controls; MCI = Mild Cognitive Impairment; DAD = Dementia due to Alzheimer’s Disease; CGM = Cortical Grey Matter; ILV = Inferior Lateral Ventricle; RAVLT = Rey-Auditory Verbal Learning Test; ICV = Intracranial volume; HOC = Hippocampal Occupancy Score.

### Convergent validity of memory tests

For the whole sample, after controlling for age and schooling, scores in RAVLT A5 strongly correlated with RAVLT A7 (r = 0.80, p<0.001) and were moderately associated with performances in Logical Memory (r = 0.45, p<0.001) and Visual Reproduction test (r = 0.47, p<0.001). Similarly, performance in RAVLT A7 was moderately related to Logical Memory (r = 0.54, p<0.001) and Visual Reproduction tasks (r = 0.56, p<0.001), whereas relationships between scores in Logical Memory and Visual Reproduction tests were also moderate (r = 0.44, p<0.001). For MCI+DAD groups, strong correlation was found between RAVLT A5 and A7 (r = 0.79, p<0.001), while a moderate association was detected between Visual Memory and RAVLT A7 (r = 0.53, p = 0.001). Those data are displayed on Table 2.

**Table 2:** Partial correlations among scores in cognitive tests and brain volumes (corrected for intracranial volumes), controlling for age and schooling.

	RAVLT A5	RAVLT A7	Logical Memory	Visual Reproduction
***Whole sample***				
*Cognitive tests*:				
**RAVLT** A5	-			
**RAVLT** A7	.80*	-		
**Logical Memory**	.45*	.54*	-	
**Visual Reproduction**	.52*	.56*	.44*	-
*Brain volumes*:				
**Left CGM**	.29	.28	.25	.34
**Right CGM**	.32	.32	.29	.41*
**Left Hippocampus**	.41*	.48*	.33	.29
**Right Hippocampus**	.41*	.46*	.34	.32
**Left ILV**	-.41*	-.45*	-.27	-.43*
**Right ILV**	-.36	-.46*	-.28	-.43*
**HOC**	.51*	.59*	.38	.40
***MCI + DAD***				
*Cognitive tests*:				
**RAVLT** A5	-	-		
**RAVLT** A7	.79*	-		
**Logical Memory**	.35	.49	-	
**Visual Reproduction**	.42	.53*	.38	-
*Brain volumes*:				
**Left CGM**	.16	.15	.23	.14
**Right CGM**	.30	.26	.28	.22
**Left Hippocampus**	.54*	.54*	.46	.32
**Right Hippocampus**	.57*	.59*	.48	.51*
**Left ILV**	-.39	-.32	-.25	-.36
**Right ILV**	-.38	-.40	-.28	-.39
**HOC**	.59*	.59*	.37	.37

*p<0.005; CGM = Cortical Grey Matter; ILV = Inferior Lateral Ventricle; RAVLT = Rey-Auditory Verbal Learning Test; HOC = Hippocampal Occupancy Score.

### Clinical-anatomical correlates of memory tests

For the whole sample, significant moderate positive correlations were found between bilateral hippocampal volumes and HOC, and RAVLT indices (A5 and A7). Left ILV moderately and negatively correlated with RAVLT and Visual Reproduction tests, whereas right ILV showed negative moderate associations with RAVLT A7 and Visual Reproduction. Right CGM related with scores in Visual Reproduction task. As for MCI+DAD group, both RAVLT A5 and A7 were moderately associated with bilateral hippocampal volumes and HOC. Right hippocampal volume was also moderately correlated with performance in Visual Reproduction. Those data are summarized in Table 2.

**Fig 1:**
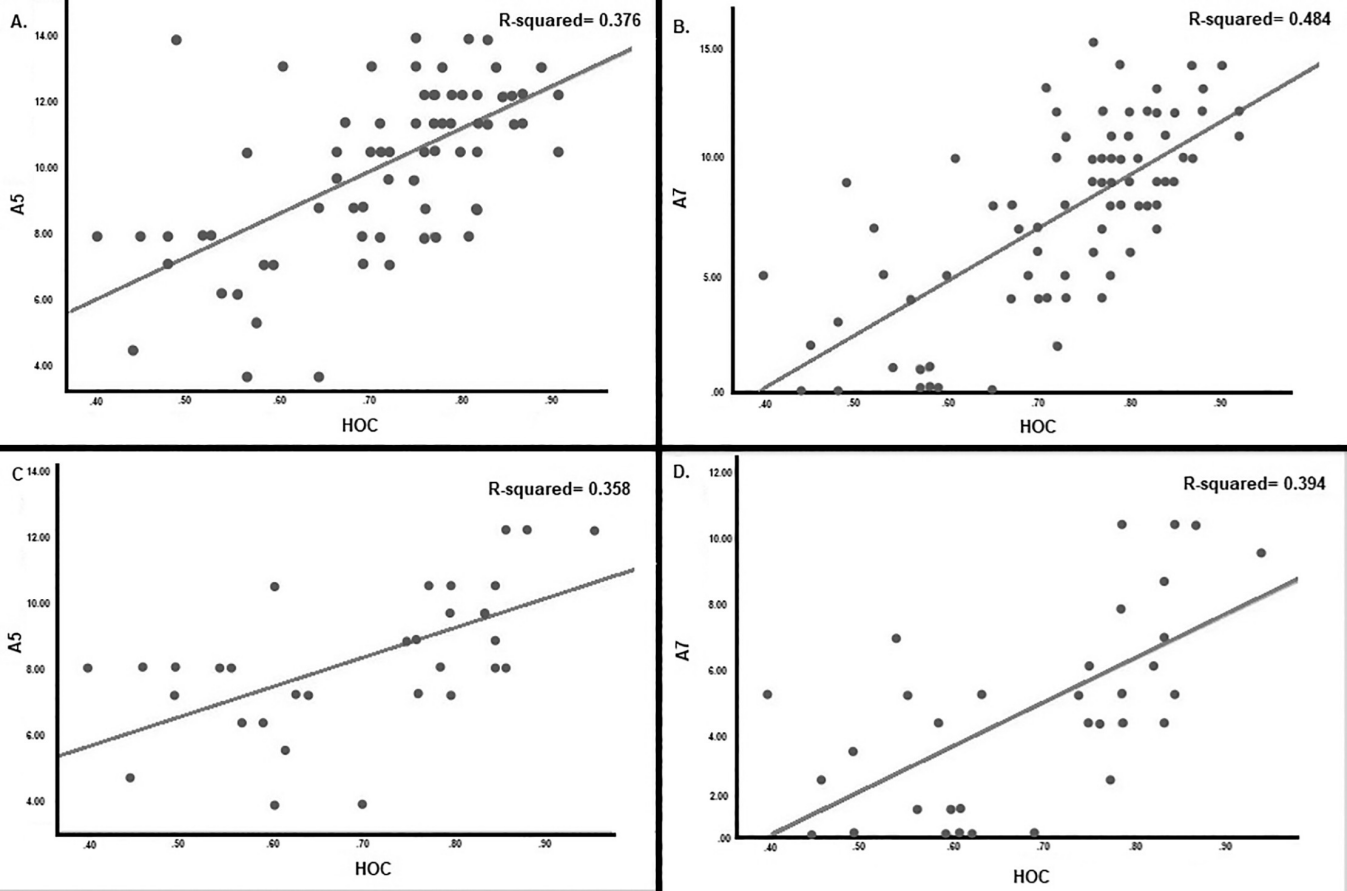
Partial correlations between RAVLT and HOC for the whole sample (A and B) and MCI+DAD (C and D).

Fig 1 illustrates partial correlations between RAVLT A5 and A7 and HOC for the whole sample (A and B) and MCI+DAD (C and D). Scores in RAVLT A5 and A7 predicted, respectively, 35–37% and 39–48% of variance in HOC volumes.

## Discussion

Performances in episodic memory tests using stimuli of different materials (verbal or visual) and displaying divergent designs (delayed recall for word lists, stories or figures) were, at most, moderately correlated in our sample, comprising controls and subjects with MCI and DAD. Likewise, scores in those tasks were also fairly associated with hippocampal and other related brain volumetric indices. Of all those measures, the RAVLT (both A5 and A7) showed the best criterion validity, exhibiting significant, yet also moderate, relationships with HOC and hippocampal volumes in subjects within the clinical spectrum of AD. RAVLT A7 predicted 39–48% of variance in HOC volumes in our sample. In contrast, delayed-recall for items of stories (assessed by Wechsler Memory Scale’s Logical Memory subtest) did not correlate with hippocampal volume measurements. Figure-learning abilities (Wechsler Memory Scale’s Visual Reproduction subtest) moderately correlated with right hippocampal volumes in MCI+AD subjects.

Given the heterogeneity of tasks designed to investigate episodic memory deficits, determining whether they could be applied interchangeably for the assessment of patients with suspected AD-related disorders, or rather, if they tap different aspects of this cognitive domain would opportunely provide guidance when defining neuropsychological protocols in clinical and research practices. Additionally, appraising the impact of distinct approaches over outcomes could be valuable for the interpretation of contrasting results across studies. In this regard, ours findings are in line with a meta-analysis addressing cognitive impairments in the AD spectrum, which indicated that discrepant effect-sizes in episodic memory performances could be explained by heterogeneities regarding neuropsychological instruments adopted in the studies[34]. Hence, considering that medial temporal atrophy has been depicted as an early biomarker of AD pathology according to a large multicenter longitudinal research[35], it could be speculated that variations on methods to assess episodic memory may have accounted to some extent for inconclusive clinical-anatomical relationships across studies.

Some other inferences regarding the definition and the neural correlates of the episodic memory construct could be drawn from our results. The lack of robust associations among cognitive tests in our study might suggest that, instead of a unitary entity, episodic memory might represent a broad overarching functional system, involving a set of distinct and loosely correlated factors. Although the number and the nature of those components remain undetermined, task- and material-specific double-dissociation frameworks have been proposed in the literature, including: verbal *versus* visuospatial memory[36], temporal *versus* spatial memory[37], recent *versus* remote autobiographical memory[38] and memory for content (“item memory”) *versus* memory for context (“source memory”)[39], among others. Those paradigms may recruit different brain circuits, such as connections of the medial temporal cortices, but also widely distributed tracts throughout the frontal, parietal and occipital lobes[36,37,39,40]. Consistently, studies assessing neurodegenerative disorders with divergent patterns of brain changes (AD *versus* Frontotemporal Lobar Degeneration and AD *versus* Parkinson’s Disease) have yielded discrepant amnestic profiles[10,41].

From this perspective, it could be accepted that differential demands regarding other cognitive abilities may also influence performances in memory tasks. Encoding and retrieval strategies applied during tests might differ according to the presence of neurocognitive disorders or to the properties of the test itself. For instance, memory for paragraph-length data (stories), as evaluated in Logical Memory test, requires contextual comprehension and semantic organization of the material[10]. In those cases, linguistic skills, semantic memory and executive function may favor associative binding of information. Appropriately, increased activation of brain areas related to working memory, such as the cingulate and the left inferior and middle frontal gyri, was detected during recollection of semantically-associated words in healthy older subjects[42]. On the other hand, encoding unrelated items from a word-list, as in the RAVLT, usually imposes more difficulty for engaging learning strategies. Hence, delayed-recall of items in word-lists has been depicted as highly dependent on the hippocampus[43], although some encoding processes have been described in the literature during this task, comprising mental imagery-creation or semantic link-inducing (for example, creating a narrative out of the words) [44,45], In addition, learning non-verbal material (as in Visual Reproduction test, for example) may also benefit from verbally recoding the stimuli[46].

Accordingly, data from a meta-analysis suggested that delayed-recall on word-lists may show higher accuracy for the diagnoses of MCI and DAD than impairments in story-learning tests[4]. It could be hypothesized whether higher demands of hippocampal-dependent processes in tasks assessing recollection of randomly unrelated set of items, as in word-lists, could account for this finding in AD subjects[47]. In contrast, recalling structured information in a story could be facilitated by relatively spared executive and language-related networks during the initial stages of the disorder. Furthermore, the relationship between scores in Visual Reproduction and the right hippocampal volumes may evoke a long-existing theory of left-right dissociation of memory systems. This disputed hypothesis implies that verbal information may depend on the left hippocampus, whereas visuospatial data may be stored within the structure in the right hemisphere[48,49].

Some limitations of the present study ought to be acknowledged. For example, since Neuroquant^®^ does not provide segmentation of prefrontal cortex, relationships among memory indices and areas associated with fronto-executive functions could not be investigated[15]. Secondly, analyses were not controlled for medication use (e.g., antidepressants, antipsychotics and anticonvulsants), which could have negatively influenced cognitive performances. In addition, language and executive function were not analyzed in this study and effects of those abilities on episodic memory were merely inferential and should be considered with caution[50]. Moreover, the small sample size did not allow testing all the different stages of episodic memory within each task (acquisition and retention) without compromising the statistical power.

## Conclusions

In conclusion, we state that different tests assessing episodic memory are not robustly correlated and should not be used interchangeably. Furthermore, performances in the RAVLT A7 significantly predicted up to 48% of the variance of the HOC volume in controls and individuals within the AD spectrum, whereas no other memory test showed similar associations with anatomical variables. Determining cognitive parameters mostly correlated with AD biomarkers might contribute for improving the characterization of the condition in clinical and research practices.
